# Temporal Profile of Descending Cortical Modulation of Spinal Excitability: Group and Individual-Specific Effects

**DOI:** 10.3389/fnint.2021.777741

**Published:** 2022-01-07

**Authors:** Jiang Xu, Alejandro J. Lopez, Maruf M. Hoque, Michael R. Borich, Trisha M. Kesar

**Affiliations:** ^1^Department of Rehabilitation Medicine, Tongji Hospital, Tongji Medical College, Huazhong University of Science and Technology, Wuhan, China; ^2^Division of Physical Therapy, Department of Rehabilitation Medicine, Emory University, Atlanta, GA, United States; ^3^Neuroscience Graduate Program, Graduate Division of Biological and Biomedical Sciences, Emory University, Atlanta, GA, United States

**Keywords:** transcranial magnetic stimulation (TMS), spinal reflex excitability, lower extremity muscles, inter-stimulus interval (ISI), corticospinal pathways

## Abstract

Sensorimotor control is modulated through complex interactions between descending corticomotor pathways and ascending sensory inputs. Pairing sub-threshold transcranial magnetic stimulation (TMS) with peripheral nerve stimulation (PNS) modulates the Hoffmann’s reflex (H-reflex), providing a neurophysiologic probe into the influence of descending cortical drive on spinal segmental circuits. However, individual variability in the timing and magnitude of H-reflex modulation is poorly understood. Here, we varied the inter-stimulus interval (ISI) between TMS and PNS to systematically manipulate the relative timing of convergence of descending TMS-induced volleys with respect to ascending PNS-induced afferent volleys in the spinal cord to: (1) characterize effective connectivity between the primary motor cortex (M1) and spinal circuits, mediated by both direct, fastest-conducting, and indirect, slower-conducting descending pathways; and (2) compare the effect of individual-specific vs. standard ISIs. Unconditioned and TMS-conditioned H-reflexes (24 different ISIs ranging from −6 to 12 ms) were recorded from the soleus muscle in 10 able-bodied individuals. The magnitude of H-reflex modulation at individualized ISIs (earliest facilitation delay or EFD and individual-specific peak facilitation) was compared with standard ISIs. Our results revealed a significant effect of ISI on H-reflex modulation. ISIs eliciting earliest-onset facilitation (EFD 0 ms) ranged from −3 to −5 ms across individuals. No difference in the magnitude of facilitation was observed at EFD 0 ms vs. a standardized short-interval ISI of −1.5 ms. Peak facilitation occurred at longer ISIs, ranging from +3 to +11 ms. The magnitude of H-reflex facilitation derived using an individual-specific peak facilitation was significantly larger than facilitation observed at a standardized longer-interval ISI of +10 ms. Our results suggest that unique insights can be provided with individual-specific measures of top-down effective connectivity mediated by direct and/or fastest-conducting pathways (indicated by the magnitude of facilitation observed at EFD 0 ms) and other descending pathways that encompass relatively slower and/or indirect connections from M1 to spinal circuits (indicated by peak facilitation and facilitation at longer ISIs). By comprehensively characterizing the temporal profile and inter-individual variability of descending modulation of spinal reflexes, our findings provide methodological guidelines and normative reference values to inform future studies on neurophysiological correlates of the complex array of descending neural connections between M1 and spinal circuits.

## Introduction

Motor evoked potentials (MEPs) generated in response to transcranial magnetic stimulation (TMS; Barker et al., 1985; Abbruzzese and Trompetto, 2002; Kobayashi and Pascual-Leone, 2003; Hallett, 2007; Kesar et al., 2018b) and Hoffman reflexes (H-reflexes) elicited in response to peripheral nerve stimulation (PNS; Schieppati, 1987; Pierrot-Deseilligny and Mazevet, 2000; Perez et al., 2007; Burke, 2016) have each been individually used to evaluate the excitability of cortical and spinal sensorimotor circuitry, respectively. Studies using TMS-evoked MEPs have shown modulation of corticospinal excitability with immobilization (Clark et al., 2008, 2010; Leukel et al., 2015; Opie et al., 2016), rehabilitation (Roosink and Zijdewind, 2010; Kantak et al., 2013; Keller et al., 2018), somatosensory stimulation (Meehan et al., 2008; Veldman et al., 2016; Brown et al., 2018), and motor learning (Stefan et al., 2005; Celnik, 2015; Kantak et al., 2018; Palmer et al., 2018). Similarly, H-reflex studies have demonstrated spinal circuit plasticity in response to electrical stimulation (Rozand et al., 2015; Bae and Kim, 2017; Kuck et al., 2018), aerobic exercise (Meunier et al., 2007; Hodapp et al., 2009; Tanuma et al., 2017), balance training (Taube et al., 2007; Behrens et al., 2015), operant conditioning (Thompson and Wolpaw, 2014, 2015) and immobilization (Lundbye-Jensen and Nielsen, 2008; Clark et al., 2010; Leukel et al., 2015). However, TMS-evoked MEPs or H-reflexes measured in isolation are limited in their ability to specifically elucidate whether the specific site of neuroplasticity is within the cortex, descending projections between M1 and the spinal cord (upper motor neurons), spinal segmental reflex circuits, or the spinal motoneuron pool, which is the final common pathway for both reflexive and voluntary motor commands (Hodgkin and Huxley, 1952; McNeil et al., 2013).

The H-reflex, providing an electrical analog of the excitability of spinal segmental reflexes, can be influenced or modulated by descending corticomotor volleys evoked by electrical or magnetic brain stimulation (Cowan et al., 1986; Nielsen et al., 1993). Therefore, the pairing of sub-threshold TMS and PNS has been used as a neurophysiologic technique to evaluate the strength of descending physiologic connections (i.e., effective connectivity) between M1 and spinal circuits (Crone et al., 2003; Urbin et al., 2017; Keller et al., 2018). TMS-conditioning of the H-reflex can index the excitability of fastest-conducting or direct as well as relatively slower or indirect descending corticomotor projections onto spinal motoneurons (Nielsen et al., 1993; Taube et al., 2017). The effect of pairing TMS with PNS manifests as a change in the amplitude of H-reflex response, when the PNS-induced ascending volley transmitted *via* the Ia afferents and TMS-induced descending volleys transmitted *via* descending corticomotor pathways converge at the level of the spinal motoneuron pool (Niemann et al., 2018). This paired, non-invasive stimulation technique can provide information about the ability of descending corticofugal pathways to modulate spinal reflex excitability in humans.

Many previous studies of TMS-conditioned H-reflexes have used two standardized inter-stimulus intervals (ISIs) to evaluate early and late interval facilitation (Nielsen et al., 1993; Nielsen and Petersen, 1995; Taube et al., 2017; Keller et al., 2018). For instance, when sub-threshold TMS is delivered 1–5 ms *after* PNS (ISI −1 to −5 ms), the resulting early onset facilitation of the H-reflex is thought to be mediated *via* direct, faster-conducting descending projections onto spinal motoneurons. When TMS is applied 5–10 ms *before* PNS (ISI +5 to +10), the resulting longer interval facilitation is hypothesized to modulate the H-reflex response through an array of relatively slower and/or indirect corticomotor descending pathways. By varying the relative timing of TMS-induced descending corticomotor volleys with respect to the PNS, varying magnitudes of H-reflex facilitation can be elicited and quantified, which in turn probe the excitability of multiple, descending pathways that influence the excitability of spinal segmental reflexes.

Previously, using a single or standard ISI, we showed moderate-to-good reliability of TMS-induced H-reflex facilitation over multiple test sessions (Gray et al., 2017). However, several methodological factors can influence the inter-individual variability and magnitudes of TMS-induced H-reflex facilitation. Previous studies have evaluated the effect of TMS intensity (Niemann et al., 2018), coil direction (Niemann et al., 2018), and muscle activation (Keller et al., 2018) on TMS-facilitation of H-reflexes. Recently, we demonstrated the effect of PNS intensity on H-reflex facilitation, albeit only at two standardized ISIs (−1.5 ms for early-onset facilitation and +10 ms for longer interval facilitation; Lopez et al., 2020). The ISI is another important but relatively under-studied parameter that can affect the magnitude and reliability of facilitation (Nielsen et al., 1993; Geertsen et al., 2011; Taube et al., 2017). Several previous studies have used a single, standardized ISI for evaluation of TMS-induced H-reflex facilitation (Gray et al., 2017; Rio-Rodriguez et al., 2017), while others determined the ISI on an individual-subject basis (Urbin et al., 2017; Niemann et al., 2018). However, the influence of ISI on the magnitude of TMS-induced modulation of H-reflexes, and inter-individual variability in the timing and magnitude of facilitation across a range of ISIs is poorly understood. In addition to inter-individual differences in conduction velocity and segment length, optimizing or individualizing the ISI for eliciting H-reflex facilitation may be particularly important in neurological conditions (e.g., stroke, spinal cord injury, multiple sclerosis) given the changes in corticospinal excitability and transmission induced by the neurological lesion or injury (Knikou, 2017; Christiansen and Perez, 2018; Li et al., 2018). Thus, as an important first step toward understanding the influence of ISI, here, we varied the ISI between TMS and PNS to systematically manipulate the relative timing of convergence of descending TMS-induced volleys with respect to ascending PNS-induced afferent volleys in the spinal cord to characterize effective connectivity between the primary motor cortex (M1) and spinal circuits, mediated by both direct, fastest-conducting, as well as indirect, relatively slower-conducting descending pathways.

Furthermore, if individualization of ISIs is indeed an important methodological consideration, determining the optimum ISI for eliciting TMS-conditioning of H-reflexes for each study participant can be tedious and time-consuming, potentially limiting or constraining the application of this paired TMS-PNS stimulation technique in clinical trials or experimental studies investigating neuroplasticity (Taube et al., 2017). Therefore, the second objective of this study was to evaluate whether using an individualized, optimal ISI presented an advantage compared to the use of the same or “standardized” ISI for all participants in the group.

## Methods

### Participants

Ten able-bodied, young individuals (eight females, age 22–28 years) participated in this study. All participants provided informed consent before study participation. Study procedures were approved by the Emory University Institutional Review Board (IRB).

### Experimental Design

**Figure 1:**
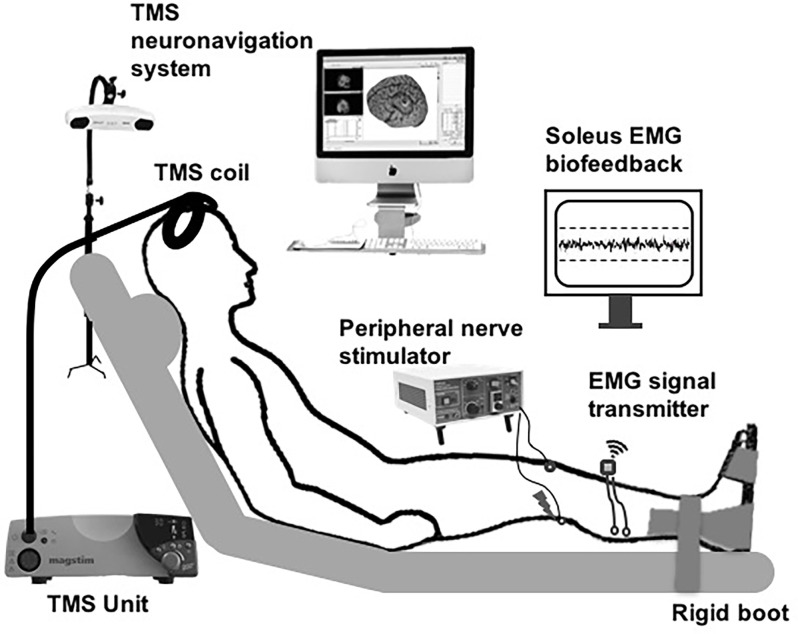
Schematic of the experimental setup. Subthreshold transcranial magnetic stimulation (TMS) pulses applied over the soleus motor cortex hotspot were paired with peripheral nerve stimulation (PNS) delivered to the tibial nerve at a range of inter-stimulus intervals (ISIs). The participants were instructed to maintain low-level background soleus muscle EMG activation targeting 10% maximal voluntary contraction (MVC), with ongoing visual biofeedback of ongoing EMG activity was displayed to the participant throughout the session.

The soleus muscle of the right leg was tested in all participants. All data-collection procedures were performed with the participants seated in a semi-recumbent position with hips and knees at 30° of flexion, and the ankles secured in rigid boots (Figure 1). The lower leg, foot, and distal thighs were stabilized with inelastic straps to maintain consistent limb positioning during the experiment.

### Electromyographic Recordings

Following standard skin preparation procedures, two surface electrodes (2-cm diameter, EL503, Biopac Systems Inc., Goleta, CA) were placed on the skin overlying the posterolateral aspect of the right soleus and the tibialis anterior (TA) muscle belly. A ground electrode was placed over the ipsilateral lateral malleolus. EMG signals were recorded at the sampling rate of 2,000 Hz with a 5–1,000 Hz bandpass filter. At the start of the experiment, participants were instructed to perform two isometric maximal voluntary contractions (MVCs) for 3–5 s into dorsiflexion and plantarflexion, separated by ~30 s of rest. To control for the effects of varying background EMG activation, the participants were requested to maintain the right soleus background EMG activity at a low-level (10% EMG activation obtained during the MVC) during data collection. Throughout the experiment, the participant was provided real-time visual biofeedback on a display screen regarding his/her ongoing average rectified soleus and tibialis anterior EMG activity, as well as the target activation level (10% MVC; Figure 1). If the participant deviated from the 10% MVC EMG activation target, the investigator would pause and instruct the participant to adjust his/her EMG activation. This EMG visual feedback and experimenter’s check on the ongoing EMG activation was implemented to ensure consistent soleus background EMG activation during the collection of both unconditioned and conditioned H-reflexes.

### Peripheral Nerve Stimulation

A square electrode (5 cm by 5 cm, TSYR2020-20, Syrtenty, Titusville, FL) was attached to the anterior aspect of the knee and served as the anode. A pen electrode was used to search for the optimal nerve stimulation site in the popliteal fossa. Electrical stimulation was delivered to the posterior tibial nerve to evoke soleus H-reflexes. The optimal site for nerve stimulation was identified as the location that elicited stable soleus H-reflexes and a visible plantarflexion contraction at higher intensities. After confirming the optimal stimulation site, a self-adherent carbon rubber circular electrode (2.5 cm diameter, TSYR1000-40 round, Syrtenty, Titusville, FL) was attached, additional pressure was applied using a Styrofoam ball to maintain the electrode’s contact with the skin, and the electrode and ball were tightly wrapped. The H-reflex recruitment curve was generated by administering approximately 50–60 single pulses with a pulse duration of 1 ms, which were delivered using an electrical stimulator controlled with custom-written MATLAB scripts (AcqKnowledge software Version 4.4, Biopac Systems Inc.). To acquire H-reflex and M-wave recruitment curves, electrical stimulation intensity was increased gradually until the maximal muscle response (M_max_) was reached, as measured by the peak-to-peak amplitude of the raw M-wave responses. Using the H-reflex and M-wave curves, we also obtained the peripheral stimulation intensity required to elicit an H-reflex amplitude of 20% M_max_.

### Transcranial Magnetic Stimulation

To elicit soleus TMS-evoked motor evoked potentials (MEPs), single TMS pulses were delivered using a custom batwing, figure-of-eight coil with a posterior-anterior current direction connected to a monophasic TMS stimulator (Magstim 200^2^; Gray et al., 2017; Kesar et al., 2018a; Figure 1). TMS pulses were delivered over the right soleus motor “hot spot” within left M1, defined as the optimal coil position that elicited maximal MEP responses in the soleus at the lowest TMS intensity. A stereotaxic neuronavigation system was used to track and maintain the accuracy of TMS coil positioning (Brainsight v. 2.2.14, Rogue Research Inc., Canada). To determine the active motor threshold (AMT), participants were requested to maintain low-level tonic EMG activity in the right soleus at 10% MVC EMG. AMT was determined as the lowest stimulator intensity needed to evoke a soleus MEP of ≥100 μV peak-to-peak amplitude in at least three out of five trials. We were able to elicit measurable MEPs from all study participants. For TMS-conditioning, the TMS intensity was maintained at 90% AMT (sub-threshold).

### TMS-Conditioning of the Soleus H-Reflex

To investigate the influence of descending corticomotor projections on spinal reflex excitability, sub-threshold (90% AMT) TMS pulses were delivered at different timing intervals, or ISIs, relative to PNS of the posterior tibial nerve. The ISI between the conditioning TMS pulse (delivered over soleus hotspot on left M1) and the test PNS pulse (delivered in the right popliteal fossa) was varied from −6 to +12 ms. We collected conditioned H-reflex data at 24 different ISIs: −6, −5, −4, −3, −2.5, −2, −1.5, −1, 0, 1, 2, 3, 4, 5, 6, 7, 8, 9, 9.5, 10, 10.5, 11, 11.5 and 12 ms. Negative ISIs indicate that the PNS was delivered prior to TMS, and positive ISIs indicate that the PNS was delivered after the TMS. At each ISI, five conditioned H-reflexes were collected, interspersed with a total of 20 unconditioned (UC) H-reflexes. PNS-evoked responses were collected at a frequency of ≤0.25 Hz in random order. The intensity of tibial nerve stimulation was set to elicit an unconditioned H-reflex peak-to-peak amplitude equivalent to 20% of M_max_, as H-reflexes of this size have been shown to be sensitive to inhibitory and facilitatory conditioning in previous publications (Taube et al., 2015, 2017; Gray et al., 2017).

### Data Processing

**Figure 2:**
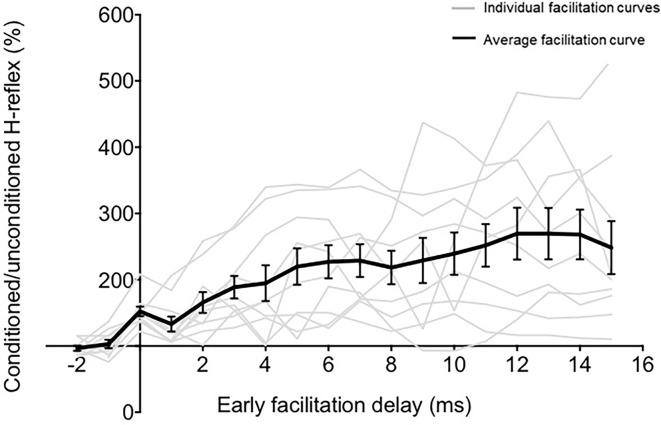
The relationship between ISI and TMS-conditioning of Hoffmann’s reflex (H-reflex). The graph demonstrates the relationship between different early facilitation delays (EFDs) and H-reflex facilitation (normalized as a percentage of unconditioned H-reflex amplitude), with gray lines representing data from individual participants and the black line representing the group average (error bars represent standard error). The ordinate (y-axis) shows the amplitude of the conditioned H-reflex as a percentage of the control (unconditioned) reflex amplitude. The abscissa (x-axis) shows the inter-stimulus timing between TMS and PNS normalized with reference to delay (in ms) from the ISI at which the earliest onset of facilitation occurred (EFD).

**Figure 3:**
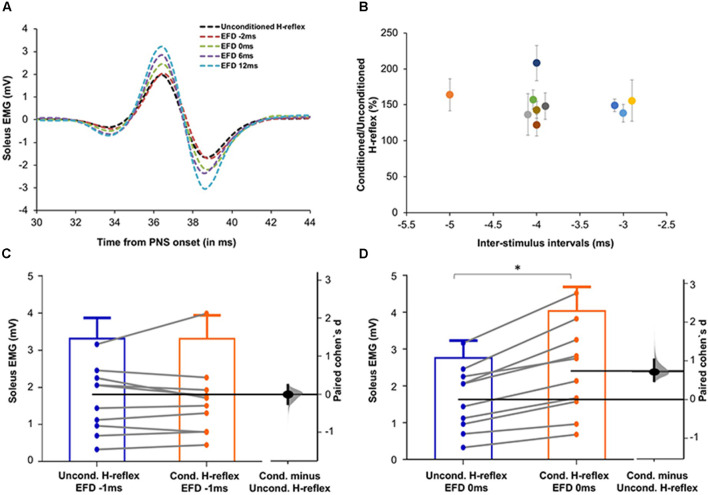
**(A)** Raw H-reflex data. Raw H-reflex traces from a representative participant without (unconditioned H-reflex) and with TMS conditioning at multiple ISIs (conditioned H-reflexes at different delays with respect to the timing of earliest onset of facilitation, i.e., EFD). The conditioned H-reflexes are displayed at EFD −2 ms, 0 ms, 6 ms, and 12 ms. Note that in contrast to the absence of facilitation at EFD 0 ms, H-reflexes at the other EFD ISI intervals are facilitated (larger in amplitude) compared to the unconditioned H-reflex, showing modulation of the spinal reflex by the descending TMS-induced volleys. **(B)** Magnitude and timing of earliest onset of facilitation. The earliest onset of facilitation was observed at ISIs ranging from −3 to −5 ms (mean = −3.70 ± 0.67ms) across study participants (x-axis). The average magnitude of facilitation (y-axis) was 158.00 ± 28.58% but varied across different ISIs. **(C,D)** Graphs with estimation plots showing comparisons between unconditioned vs. conditioned H-reflex amplitudes at EFD −1 ms and EFD 0 ms. Unconditioned and conditioned H-reflexes (means with standard deviation as well as individual participant data) are shown at EFD −1 ms **(C)** and EFD 0 ms **(D)**. The paired mean difference (Cohen’s d) is shown with a Gardner-Altman estimation plot on a floating axis on the right as a bootstrap sampling distribution; the mean difference is depicted as a dot; the 95% confidence interval is indicated by the ends of the vertical error bars. Note that while no significant increase in the conditioned H-reflex amplitudes was observed at EFD −1 ms, a significantly larger amplitude of conditioned vs. unconditioned (with a large effect size) was observed at EFD 0 ms. *Indicates statistically significant difference.

Peak-to-peak amplitudes of unconditioned and conditioned H-reflexes at each ISI were extracted from raw EMG recorded. At each ISI, the magnitude of H-reflex facilitation or modulation was calculated as conditioned H-reflex amplitude as a percentage of the unconditioned H reflex amplitude. The earliest onset of facilitation indicates the ISI at which the fastest descending TMS-induced volleys arrive at the spinal motoneuron pools. The ISI of onset of earliest facilitation was identified for each participant as the first peak of facilitation that was followed by a period of decline before the facilitation curve resumed (Nielsen et al., 1993; Taube et al., 2015, 2017; Niemann et al., 2018; Figure 2). We plotted the relationship between ISI (normalized to EFD 0 ms) and the magnitude of H-reflex facilitation for each participant (Figure 2). Once we determined the onset of earliest facilitation, the ISI of this time point was redefined as 0 ms, and all previous and subsequent ISIs were normalized with reference to the earliest onset of facilitation, described as early facilitation delays (EFDs; Figure 2). We statistically compared unconditioned vs. conditioned H-reflex amplitudes at EFD 0 ms, as well as at EFD −1 ms to confirm that a significant facilitation occurred at EFD 0 ms (Figure 3).

In addition to the individualized determination of the onset of earliest facilitation and magnitude of facilitation at a range of EFDs, the individual-specific peak facilitation for each participant was determined at the ISI that generated maximal facilitation. Previous studies have defined standard early and longer interval or late ISIs as −1.5 ms and +10 ms, respectively. To compare facilitation observed at individualized (determined for each individual using their ISI curve) vs. standard ISIs (the same ISI used for all participants), we compared the magnitude of facilitation at: (i) individualized ISI where earliest facilitation was observed (EFD = 0 ms) vs. a standardized early ISI of −1.5 ms; and (ii) individualized maximal facilitation vs. longer interval facilitation at a standardized ISI of +10 ms.

### Statistical Procedures

A repeated-measures analysis of variance (ANOVA) was performed to evaluate the effect of EFD (within-subjects factor with 18 levels, from EFD −2 ms to EFD 15 ms) on the dependent variable of H-reflex facilitation. We used paired t-tests to compare unconditioned vs. conditioned H-reflex amplitudes at EFD 0 ms and at EFD −1 ms. Additionally, to evaluate conditioning effects at standardized vs. individualized ISIs, paired t-tests were used to compare the magnitude of facilitation at: (i) the individual-specific ISI where the earliest onset of facilitation occurred (EFD 0 ms) vs. at a standard early ISI of −1.5 ms; (ii) the individual-specific maximal or peak facilitation vs. at a standard longer interval ISI of +10 ms. The Shapiro-Wilks test showed normal distribution at all except one ISI (EFD = 1 ms). All statistical tests were run in Statistical Package for the Social Sciences (IBM SPSS version 26) and the critical alpha level was set to *p* < 0.05.

## Results

### Identification of the Earliest Onset of H-Reflex Facilitation in Individual Participants

**Figure 4:**
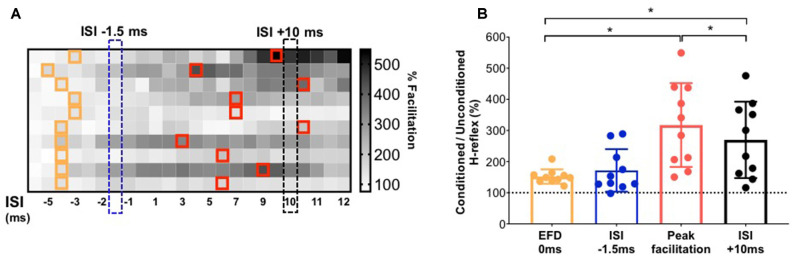
Demonstration of individualized earliest onset of and peak magnitude of facilitation in comparison to standard ISIs. **(A)** The gradient map shows the gray-scale rank of the magnitude of facilitation for each study participant (rows) at each ISI (columns). The cells in the map filled with different gray scale colors (from white to black) represent the rank of conditioned H-reflexes amplitude (from highest facilitation to lowest facilitation) for each participant. For each participant, ISIs at which the earliest onset of facilitation was detected are demarcated with orange outlines. On the same color matrix, individualized ISIs that elicited peak facilitation are demarcated with red outlines. The chart shows the inter-individual variability in the location of ISI that elicits earliest or peak facilitation. **(B)** Comparison of the magnitude of facilitation for early facilitation at individualized EFD 0 ms vs. at a standard early ISI of −1.5 ms; and between individualized peak facilitation vs. at a longer interval standard ISI of +10 ms. *Indicates statistically significant difference.

To determine the influence of TMS-induced fastest descending volleys arriving at the spinal motoneurons, we identified the earliest onset of H-reflex facilitation for each participant. Relationships between ISI and H-reflex facilitation for each participant are shown in Figure 2. The onset of the earliest facilitation was observed at ISIs ranging from −3 to −5 ms (mean = −3.70 ± 0.67 ms) across study participants (Figure 3B). The average magnitude of facilitation at the earliest onset of facilitation was 158.00 ± 28.58% (Figures 2, 4).

Each individual’s data were normalized with reference to the ISI of earliest onset, which was referred to as EFD 0 ms). To confirm that EFD 0 ms was the first arrival of the fastest descending volley at spinal circuits, we statistically compared the unconditioned and conditioned soleus H-reflex amplitudes at EFD −1 ms and 0 ms (Figures 3C,D). At EFD −1 ms, there was no significant difference between the unconditioned and conditioned H-reflex amplitude [*p* = 0.99, paired Cohen’s d −0.00056 (95.0%CI −0.262, 0.246)]. At EFD 0 ms, there the conditioned H-reflex amplitude was significantly larger than unconditioned [*p* = 0.0018, paired Cohen’s *d* = 0.709 (95.0%CI 0.471, 1.04)] (Figure 3).

### Influence of ISI on TMS-Conditioning of H-Reflex Facilitation

Overall, sub-threshold TMS conditioning facilitated the H-reflex from EFD 0 ms to EFD +15 ms (Figure 2). The repeated measures ANOVA evaluating the effect of EFD on H-reflex facilitation revealed a significant main effect for EFD (F_2.815,25.335_ = 8.406, *p* < 0.01, η^2^ = 0.483).

### Comparison of Early and Late Facilitation Measured at Individualized ISI vs. Standard ISI

To facilitate an individual-specific visualization of our study results, the magnitudes of H-reflex facilitation for each study participant (rows) at each ISI (columns) are demonstrated as a gray-scale gradient map (Figure 4A), with the different gray scale colors (from black to white) representing the rank of conditioned H-reflex amplitudes (from highest facilitation to lowest facilitation) for each participant. The onset of earliest facilitation and peak facilitation are both demarcated in the map for each participant, showing the inter-individual variability in these ISIs (Figure 4A). The paired t-test showed no significant difference between the facilitation magnitude at the individualized ISI where earliest onset facilitation was observed (EFD 0 ms) vs. facilitation at the standard early ISI of −1.5 ms [152.20 ± 23.05%, 171.78 ± 68.29%, *p* = 0.30, paired Cohen’s *d* = 0.384 (95.0%CI −0.289, 1.1)] (Figure 4B). In contrast, the paired t-test showed a significantly greater magnitude of peak H-reflex facilitation when determined using an individualized peak ISI vs. the standard longer interval ISI of +10 ms [317.51 ± 134.54%, 269.83 ± 122.57%, *p* < 0.01, paired Cohen’s d −0.332 (95.0%CI −0.515, −0.204)] (Figure 4B). The magnitude of peak facilitation at longer interval ISIs (both using individualized peak and standard ISI of +10 ms) was significantly greater than the magnitude of earliest onset facilitation observed at EFD 0 ms (*p* < 0.01; Figure 4B).

## Discussion

Here, we studied the temporal dynamics underlying pairing of TMS with PNS, a promising, non-invasive neurophysiologic approach for indexing the excitability of both direct or fast and indirect or relatively slower descending corticomotor projections onto spinal segmental circuitry. We systematically quantified the inter-individual variability in both the magnitude and timing of occurrence (i.e., ISI) of the earliest onset and peak TMS-induced H-reflex facilitation. Our results revealed substantial inter-individual variability in the timing of the earliest onset and the greatest magnitude of H-reflex facilitation, and support potential methodological advantages and mechanistic insights gained from utilizing individualized ISIs for measurement of earliest onset and longer-interval facilitation.

### Mechanisms and Interpretation of Earliest Onset of Facilitation

Our findings show that the earliest onset of H-reflex facilitation occurred at the ISI of −3.70 ± 0.67 ms in healthy participants (Figures 2, 3). The negative values for ISIs of early facilitation indicate that the TMS pulse was delivered after the PNS pulse. This earliest onset of facilitation is posited to represent the excitability of the fastest conducting or direct descending corticomotoneuronal connections (Nielsen et al., 1993; Taube et al., 2015, 2017). In our study, at these ISIs, the TMS-induced volley in these fastest or direct descending pathways produced sufficient depolarization in spinal motoneuron pools innervating the soleus, as evidenced by the larger amplitude of the conditioned H-reflex compared to an unconditioned H-reflex (Leukel et al., 2015; Taube et al., 2015). We identified the earliest onset of facilitation from the ISI curve as the first peak of facilitation followed by a decline in facilitation, based on methodology from previous literature (Keller et al., 2018; Figure 2). The decline following the first peak, while poorly understood, may be mediated by the activation of spinal inhibitory interneurons (Nielsen et al., 1993). In our study, the ISI at which we observed earliest facilitation is consistent with findings from a previous study that showed that early-onset or short-latency facilitation occurred in the −4 to −2 ms (−3.54 ± 0.66 ms) ISI range when the soleus was at rest (Taube et al., 2015, 2017). Another study, which used early-onset facilitation to evaluate the effects of ankle joint immobilization, revealed that early facilitation occurred at around the −3 ms ISI on average (Leukel et al., 2015). Similar to our current methods, a previous study recalibrated the ISI of early facilitation as 0 ms to synchronize the subsequent ISIs with respect to the ISI eliciting the earliest onset of facilitation (Aguiar and Baker, 2018). Based on our findings, future studies interested in evaluating the excitability of fastest and/or direct conducting descending projections can test ISIs in the −3 to −4 ms range, normalize individual ISI curves to the ISI of onset of earliest facilitation (EFD 0 ms), and to improve accuracy, if time permits, individually determine the ISI at which early facilitation is observed.

Although several previous studies used one standard ISI to investigate early H-reflex facilitation in their study cohort (Cortes et al., 2011; Leukel et al., 2012; Gray et al., 2017), early facilitation may show inter-individual variability due to physiological and anatomical differences influencing conduction velocities. In our study participant cohort, if we selected a single or standard ISI in the early facilitation range, a subset of participants may not demonstrate H-reflex facilitation (i.e., facilitation greater than 100%). Thus, in addition to evaluating the magnitude and timing of the earliest onset of facilitation, determining the individualized ISI that elicits the greatest magnitude of facilitation can be advantageous. For instance, the magnitude of earliest onset facilitation (i.e., % modulation of the conditioned H-reflex amplitude at EFD 0 ms) would provide a measure of the strength of effective connectivity of the fastest conducting and/or direct descending pathway between M1 and the spinal motoneuron pool. In conjunction, the ISI at which the earliest onset of facilitation occurs (i.e., ISI at which EFD 0 ms occurs) may provide insights into the underlying mechanism of descending cortical influences on spinal excitability. For example, if EFD 0 ms occurs at a longer ISI, showing a rightward shift with a neuropathology, it could indicate that the weakened effective connectivity is in part caused by delayed conduction or aberrant transmission in this fastest and/or direct descending pathway. Interestingly, recent work utilized shorter-interval ISIs with greater temporal resolution than our study (up to 0.1 ms differences between ISIs), and inferred that EFD 0 ms and EFD +0.6 ms were informative of changes in the excitability of circuits within infragranular and supragranular cortical layers, respectively (Leukel and Kurz, 2021).

Our results showing no significant difference in the measured magnitude of early facilitation when using a standardized (−1.5 ms) vs. individualized ISI suggest that at least for younger able-bodied individuals (Figure 4), the measurement of earliest onset of facilitation is robust and perhaps less susceptible to inter-trial or physiological variability. The Early onset facilitation measured at a single, standardized ISI such as −1.5 ms may provide a relatively quick and useful index of the overall excitability of relatively fast and direct descending projections. The statistically significant facilitation of the conditioned H-reflex measured at the −1.5 ms ISI also suggests that the coincidence of TMS-induced descending and PNS-induced ascending afferent volleys at the spinal motoneurons elicit robust H-reflex facilitation in able-bodied individuals, and probe the overall excitability or effective connectivity in the population of direct and/or fast descending projections (Cortes et al., 2011; Leukel et al., 2012).

### Mechanisms and Interpretation of Longer-Interval and Maximal Facilitation

In addition to evaluating the timing and magnitude of the earliest onset of TMS-induced H-reflex facilitation, we also evaluated a wide range of longer interval ISIs and determined the ISI that elicits the greatest facilitation in our young able-bodied participant cohort (Figure 2). As expected, our results showed that the magnitude of peak facilitation was significantly higher than that of earliest onset facilitation (EFD 0 ms). We also showed a high degree of inter-individual variability in the individualized ISIs that elicited peak magnitude of facilitation (Figure 4A). This inter-individual variability in the magnitude and ISI of occurrence of peak facilitation perhaps reflects the complex and varied array of descending projections that may contribute to the facilitation measured at longer interval ISIs. The longer interval or late facilitation is hypothesized to be mediated by polysynaptic and/or relatively slower conducting, corticofugal descending pathways between M1 and the spinal motoneuron pool. While this method only provides indirect inference and limited anatomic specificity of these descending pathways, previous studies have hypothesized that they include cortico-reticulo-spinal, cortico-vestibular-spinal, cortico-propriospinal, and spinal interneurons that can synapse on multiple populations of spinal interneurons and motoneurons (Nielsen et al., 1993; Serranova et al., 2008).

In our study, similar to some previous works, we utilized the ISI of +10 ms as the standardized group ISI to measure longer interval facilitation. The challenge with the paired TMS-PNS technique, especially during the measurement of longer interval facilitation, is that multiple descending volleys may contribute additive EPSPs onto spinal motoneuron membranes, providing a varied spatial and temporal integration of membrane excitability at the spinal motoneuron pool, which could also be a potential explanation for the higher magnitude of facilitation observed at the longer interval ISIs. The conduction velocities and number of synapses in corticomotor projections may vary across individuals (Nielsen et al., 1993; Serranova et al., 2008). Also, the polysynaptic and multi-pathway neural circuit correlates underlying longer interval facilitation may result in greater variability in amplitudes of conditioned soleus H-reflexes (Nielsen et al., 1993; Nielsen and Petersen, 1995; Gray et al., 2017). The TMS-induced descending volleys within different corticofugal pathways may contribute to the inter-individual differences in both the ISI at which peak facilitation is observed and the magnitude of peak facilitation. Our results that a significantly larger magnitude of peak facilitation was observed at individualized ISIs vs. a standard 10 ms ISI, as well as the inter-individual variability in the ISI at which peak facilitation was observed, suggest that individualized ISIs may need to be determined for a comprehensive and rigorous measurement of TMS-induced H-reflex facilitation mediated by slower and/or indirect descending pathways.

Based on our current results, we posit that the measurement of longer-interval facilitation (e.g., EFD 0 ms to 12 ms) at a range of ISIs can help to index the effective connectivity of relatively slower and/or indirect pathways. If the methodological or time constraints of a study necessitate a brief assessment of descending corticospinal effective connectivity, then peak facilitation at longer interval ISIs, even at a single ISI (e.g., +10 ms) may be sufficient to index the cumulative strength or excitability of the array of other descending connections between M1 and spinal motoneurons that are distinct from the fastest and/or direct descending pathways probed *via* earliest-onset facilitation. For a more refined or fine-grained characterization of descending corticospinal effective connectivity, a comprehensive longer-interval ISI curve for individual subjects (similar to Figure 3) can be collected, and also used to map changes in the ISI curve before vs. after an intervention or experimental manipulation. If the ISI vs. H-reflex facilitation curve shifts upward at longer intervals, that may suggest an increased effective connectivity in the slower or indirect descending pathways. If the peak or the entire curve shifts to the right, this may indicate a greater and perhaps compensatory reliance on a sub-population of relatively slower or indirect pathways (e.g., brain stem or propriospinal-mediated descending projections).

### Potential Mechanisms and Implications of Inter-Individual Variability in Magnitude and Timing of TMS-Induced Facilitation

We posit that a neurophysiological assessment battery combining TMS, H-reflexes, and TMS-conditioned H-reflexes can probe site-specific changes in descending corticomotor circuits (Kurz et al., 2019), spinal reflex circuits (Niemann et al., 2018), and interactions between the two. Thus, utilization of paired TMS and PNS can provide more in-depth mechanistic insights into the specific site(s) and magnitude of training-induced plasticity in sensorimotor control circuitry. TMS-derived measures (e.g., MEP amplitude, motor threshold) and H-reflex data can be influenced by testing conditions such as posture, muscle activation, EMG sensor or stimulation electrode position, etc. In the current study, we controlled for and maintained consistency of, these methodological parameters, manipulating only the relative timing of delivery of TMS with respect to PNS, and collecting unconditioned H-reflexes as a control or reference for the TMS-conditioned H-reflex amplitudes. Inter-individual differences in H-reflex and MEP latencies, nerve conduction velocity, limb length, neuroanatomical structure, and strength of effective functional connectivity may explain the variability observed in the magnitude of facilitation as well as the ISIs eliciting earliest or peak facilitation. Additionally, for the same individual, the trial-to-trial variability in latencies and relative synchronization of TMS-induced descending volleys and PNS-induced ascending volleys may result in physiological variability in the arrival time of orthodromic and antidromic stimuli at the spinal motoneurons (Baudry et al., 2015), which can further contribute to the variability in TMS-induced facilitation. In recent work by Wiegel and Leukel, during TMS-conditioning of H-reflexes, different TMS intensities (e.g., above resting motor threshold), as well as transcranial electrical stimulation, were used in upper limb muscles to characterize different cortical pathways (Leukel and Kurz, 2021). Recent work in monkey and human models also underscores the possibility of measuring the excitability of different cortical circuits by TMS H-reflex conditioning (Wiegel et al., 2018; Kurz et al., 2019).

### Limitations and Future Directions

The limitations of this study include the relatively small sample size, although it is consistent with samples in other similar studies (Gray et al., 2017; Lopez et al., 2020; Capozio et al., 2021). For the current study, a single nerve stimulation intensity was chosen (PNS intensity eliciting an H-reflex amplitude of 20% Mmax) based on previous literature, and facilitation was not measured at a range of intensities across the H-reflex recruitment curve. Similarly, a single subthreshold TMS intensity was used. For instance, in recent studies, H-reflex facilitation at certain EFDs was shown to be differentially influenced by specific movement tasks (Wiegel et al., 2020; Wiegel and Leukel, 2020). The stimulation parameters used here (e.g., 1 ms pulse width, the separation between consecutive ISIs, sub-threshold TMS according to active motor threshold), while mostly consistent with previous work, have been modified in recent studies, particularly to probe specific neural contributions at the ISIs eliciting early facilitation. Future studies can investigate factors influencing the optimal ISI (e.g., the latency of MEPs and H-reflexes) and develop a formula to enable individual-specific estimation of the optimal ISI for measuring earliest onset and peak facilitation.

Although earliest-onset and peak facilitation derived using paired TMS and PNS help to better localize the site of plasticity compared to TMS alone or H-reflexes alone, these techniques do not have the specificity to identify exactly which neural pathway is implicated in eliciting facilitation at different ISIs. As is true for many other non-invasive approaches, especially at longer ISIs, the observed H-reflex facilitation can be caused by many neural sources, spanning spinal, brain stem, and cortical sites, which are challenging to discriminate. Future work can combine TMS-facilitation of H-reflexes with neuroanatomical imaging or complementary neurophysiological techniques to determine relative contributions of specific descending pathways (e.g., cortico-reticulo-spinal, proprio-spinal) to these measures. Further, this method is limited to those muscles from which H-reflexes can be consistently elicited. The soleus and other lower limb muscles may have stronger spinal network contributions, which could influence our findings. Between-muscle differences in neuromotor circuit control can be investigated by applying similar methods in upper limb muscles and other lower limb muscles. We measured H-reflex facilitation in a seated active state for our study (i.e., while participants maintained low-level background EMG of ~10% MVC); future work can compare facilitation in seated vs. standing, or during a dynamic postural or walking task (Nielsen et al., 1993; Nielsen and Petersen, 1995).

Evaluation of the effect of neuropathologies such as stroke or spinal cord injury on the magnitude and temporal profile of TMS-induced H-reflex facilitation is a promising area of future investigation. Understanding how the relationship between ISI and magnitude of H-reflex facilitation (i.e., the ISI curve similar to shown in Figure 1) is modulated by neuropathological conditions warrants more study. For example, one may postulate that in individuals with a cortical or subcortical lesion affecting the corticospinal pathway, the faster-conducting, direct descending projections may show greater disruption of effective connectivity compared to relatively slower, indirect descending projections that traverse through brain stem centers (Li and Francisco, 2015; Li et al., 2018). In fact, these relatively slower and indirect descending pathways, partly indexed using peak facilitation or area under the curve for longer-latency ISIs, may show a compensatory reorganization to mediate functional recovery following stroke (Wilkins et al., 2020; Hammerbeck et al., 2021). We therefore would hypothesize that post-stroke individuals may show a greater or preferential reduction in the magnitude of earliest onset of TMS-induced facilitation (i.e., EFD 0 ms).

## Conclusions

Our study provides further evidence showcasing the advantage of TMS-induced H-reflex facilitation, especially when measured at a range of ISIs, as a unique non-invasive probe to differentially parse out the excitability of the array of direct, fast and indirect, slower descending corticomotor projections onto spinal reflex circuits. Our study findings can guide the methodology for use of the paired TMS-PNS technique in future investigations. Due to variability in conduction latencies of neuronal tracts in neurologically impaired individuals (such as stroke or multiple sclerosis), as well as inter-individual variability in physiological latencies, further development of methods and formulae to estimate the optimal ISI between TMS and PNS for each individual based on their baseline data merits further investigation.

## Data Availability Statement

The raw data supporting the conclusions of this article will be made available by the authors, without undue reservation.

## Ethics Statement

The studies involving human participants were reviewed and approved by Emory University Institutional Review Board (IRB). The patients/participants provided their written informed consent to participate in this study.

## Author Contributions

TK and MB conceived and designed research. MH performed experiments with a group of graduate students. JX, AL, and MH analyzed data, prepared figures, and drafted manuscript. JX, AL, TK, MB, and MH interpreted results of experiments, prepared, edited and revised the manuscript, and approved the final version of manuscript. TK edited the final versions of the manuscript. All authors contributed to the article and approved the submitted version.

## Conflict of Interest

The authors declare that the research was conducted in the absence of any commercial or financial relationships that could be construed as a potential conflict of interest.

## Publisher’s Note

All claims expressed in this article are solely those of the authors and do not necessarily represent those of their affiliated organizations, or those of the publisher, the editors and the reviewers. Any product that may be evaluated in this article, or claim that may be made by its manufacturer, is not guaranteed or endorsed by the publisher.

## Abbreviations

TMS: Transcranial magnetic stimulation
PNS: Peripheral nerve stimulation
ISIs: Inter-stimulus intervals
EFD: Early facilitation delay
EPSPs: Excitatory postsynaptic potentials
MVC: Maximal voluntary contraction
AMT: Active motor threshold
ANOVA: Analysis of variance
